# Burden of Depression and Its Determinants Among Primary Caregivers of Individuals With Severe Mental Illness in Uganda: A Multicenter Cross‐Sectional Study

**DOI:** 10.1002/hsr2.72558

**Published:** 2026-05-25

**Authors:** Florent Ishimwe, Marie Pascaline Sabine Ishimwe, Maxwell Okello, Theodore Nteziyaremye, Alinaitwe Racheal, Usman Ibe Micheal, Joshua Muhumuza, Ildephonse Dushimirimana, Musinguzi Ronald, Abdirazak abdinazir, Kiswezi Kazigo Ahmed, Norbert Gumisiriza, Theoneste Hakizimana

**Affiliations:** ^1^ Department of Mental Health and Psychiatry Kampala International University Ishaka Uganda; ^2^ Department of Pediatrics and Child Health Kampala International University Ishaka Uganda; ^3^ Department of Obstetrics and Gynecology Kampala International University Ishaka Uganda; ^4^ Department of Sciences University of Rwanda Kigali Rwanda; ^5^ Department of Surgery Kampala International University Ishaka Uganda

**Keywords:** depression, perceived stigma, primary caregivers, severe mental illness, social support, Uganda

## Abstract

**Background and Aims:**

Primary caregivers of individuals with severe mental illness (SMI) often experience substantial psychological distress, yet evidence from Uganda remains limited. This study aimed to determine the prevalence of depression and its associated factors among primary caregivers of individuals with SMI in Uganda.

**Methods:**

We conducted a multicenter cross‐sectional study among 376 consecutively recruited primary caregivers of individuals with SMI attending Jin[ja Regional Referral Hospital and Mbarara Regional Referral Hospital. Data were collected using interviewer‐administered questionnaires, including the Patient Health Questionnaire‐9 for depression, the devaluation of consumer families scale for perceived stigma, and the multidimensional scale of perceived social support for social support. Data were analyzed using SPSS version 22.0. Multivariable logistic regression was used to identify factors independently associated with depression, and adjusted odds ratios (aORs) with 95% confidence intervals (CIs) were reported.

**Results:**

The prevalence of depression among primary caregivers was 37.8% (142/376; 95% CI: 32.7%–42.8%). Among caregivers with depression, 99 (69.7%) had moderate depression, 35 (24.7%) had moderately severe depression, and 8 (5.6%) had severe depression. Depression was independently associated with high perceived stigma (aOR = 10.29, 95% CI: 3.16–33.50, *p* < 0.001), low perceived social support (aOR = 10.51, 95% CI: 2.95–37.45, *p* < 0.001), caring for patients aged 18–44 years (aOR = 4.20, 95% CI: 1.30–13.58, *p* = 0.017), caring for patients aged 65 years or older (aOR = 8.38, 95% CI: 1.50–46.89, *p* = 0.016), and caring for patients with major depression (aOR = 7.45, 95% CI: 2.76–20.14, *p* < 0.001).

**Conclusion:**

Depression was common among primary caregivers of individuals with SMI in this Ugandan multicenter study. Higher perceived stigma, lower perceived social support, and selected patient characteristics were associated with caregiver depression. These findings highlight the need for caregiver‐focused mental health screening and psychosocial support within routine psychiatric care.

**Abbreviations**
DCFS: devaluation of consumer family's scale
JRRH: Jinja regional referral hospital
KIU‐TH: Kampala international university‐teaching hospital
MRRH: Mbarara regional referral hospital
MSPSS: multidimensional scale of perceived social support
PHQ9: patient health questionnaire‐9
PSMI: patients with severe mental illnesses
REC: research ethics committee

## 1 Introduction

Depression is a common and disabling mental health condition that affects an estimated 280 million people worldwide [1]. It is characterized by persistent low mood, loss of interest or pleasure, and a range of cognitive, emotional, and physical symptoms that impair daily functioning and overall well‐being [2]. Beyond its direct psychological effects, depression is also associated with excess morbidity and mortality, including through its links with chronic medical conditions such as cardiovascular disease, cancer, and diabetes mellitus [3].

Primary caregivers of individuals with mental illness, particularly severe mental illness (SMI), are especially vulnerable to depression and other forms of psychological distress. Caregiving in this context often extends far beyond routine support and may involve prolonged supervision, management of challenging behaviors, financial strain, and disruption of family and social life [4]. Previous work has suggested that depression among caregivers of individuals with mental illness may be more than twice as common as in the general population [5]. Caregiving has also been associated with poorer physical health, reduced quality of life, and substantial interference with employment, social participation, and daily activities [6, 7].

The risk of depression among caregivers is shaped by a range of caregiver‐, patient‐, and context‐related factors. Previous studies have linked caregiver depression to younger age, lower educational attainment, exposure to patient aggression, perceived stigma, prolonged caregiving hours, and inadequate social support [7, 8, 9, 10]. Patient‐related factors such as chronicity of illness and repeated psychiatric hospitalization may further intensify the burden of caregiving [4]. Over time, caregivers often reorganize their lives around the needs of the affected individual, which may compromise their social, occupational, and family functioning and increase vulnerability to depressive and anxiety symptoms [11, 12].

Despite their central role in the care of individuals with SMI, caregivers are frequently overlooked within mental health systems and are often treated as an informal, unpaid extension of care. This challenge has become even more important in the context of deinstitutionalization, where individuals with mental illness spend less time in hospitals, and more of their long‐term care is shifted to families and communities [13]. Although community‐ and family‐based care may improve continuity of support for patients, it can also impose substantial emotional, social, and financial demands on caregivers [11].

In low‐resource settings such as Uganda, these pressures may be amplified by limited mental health infrastructure, few structured rehabilitation services, and the absence of strong social protection mechanisms for affected families. As a result, family members often become the primary and sometimes only source of care for individuals with SMI. While this caregiving role is essential for supporting treatment adherence, reducing relapse, and maintaining day‐to‐day functioning, it may come at considerable psychological cost to caregivers themselves [13].

Although caregiver mental health is increasingly recognized as an important public health issue, empirical evidence from Uganda remains limited, particularly from multicenter studies that capture variation across settings. Generating such evidence is important for informing caregiver‐focused mental health services and strengthening family support within psychiatric care. This study, therefore, aimed to determine the prevalence of depression and its associated factors among primary caregivers of individuals with SMI in Uganda using a multicenter cross‐sectional design.

## 2 Materials and Methods

### 2.1 Study Design and Setting

This hospital‐based multicenter cross‐sectional study was conducted between April 2024 and July 2024 among caregivers of PSMI at the mental health units of Jinja Regional Referral Hospital (JRRH) and Mbarara Regional Referral Hospital (MRRH). JRRH and MRRH are the busiest referral hospitals in eastern and western Uganda, respectively. They offer comprehensive mental health services with staff of different cadres, including nurses, psychologists, and psychiatrists in the mental health department.

### 2.2 Study Population

This study included primary caregivers of patients with SMI attending the mental health units of Jinja Regional Referral Hospital and Mbarara Regional Referral Hospital. Eligible participants were caregivers aged 18 years and above who were directly involved in the patient's care and who consented to participate. Caregivers who had been absent from caregiving responsibilities for more than 1 month in the previous 6 months were excluded.

### 2.3 Sample Size Determination

The sample size was calculated using Daniel's formula, Daniels WW, 1999

$$N=\frac{Z^{2}p(1-p)}{e^{2}}$$

Where *N* is the required sample size estimate, and *Z* is the critical value for a normal distribution at the 95% confidence level, corresponding to 1.96. *p *= estimated prevalence of depression among caregivers of PSMI, which was 53.5% according to a study performed in China [14, 15], *q* = 1 − *p;* therefore, $N=\frac{1.96^{2}*0.535(1-0.535)}{0.05^{2}}=376$

Therefore, the minimum required sample size was 376 caregivers of PSMI.

### 2.4 Sampling Technique

Eligible primary caregivers of individuals with severe mental illness receiving care at Jinja Regional Referral Hospital and Mbarara Regional Referral Hospital were recruited consecutively until the required sample size was reached. Each caregiver was screened for eligibility, given information about the study, and enrolled only after providing written informed consent. Recruitment was conducted at both study sites throughout the study period until the target sample size was achieved. Following enrollment, each participant completed a structured interviewer‐administered questionnaire to collect sociodemographic and psychological data.

### 2.5 Study Procedure and Data Collection Instruments

Primary caregivers of individuals with severe mental illness who attended the mental health units of Jinja Regional Referral Hospital and Mbarara Regional Referral Hospital during the study period were screened for eligibility and approached consecutively for recruitment. After a detailed explanation of the study, written informed consent was obtained in English or the local language from those who agreed to participate. Interviews were conducted in a private space within the hospital to ensure confidentiality. Data were collected using a structured interviewer‐administered questionnaire covering sociodemographic and psychological characteristics. Depression was assessed using the Patient Health Questionnaire‐9 (PHQ‐9), perceived social support using the Multidimensional Scale of Perceived Social Support (MSPSS), and perceived stigma using the Devaluation of Consumer Families Scale (DCFS). All completed questionnaires were checked for completeness and consistency before being stored securely for data management and analysis.

### 2.6 Study Variables

The independent variables in this study included selected caregiver‐ and patient‐related factors that could be associated with depression among primary caregivers of individuals with severe mental illness. Caregiver‐related variables included age, sex, residence, marital status, education level, employment, monthly income, relationship to the patient, past history of mental illness, perceived stigma measured using the devaluation of consumer families scale, and perceived social support measured using the multidimensional scale of perceived social support. Patient‐related variables included age, sex, marital status, education level, employment, monthly income, diagnosis, suicidal ideation, number of admissions, and medication compliance.

The dependent variable was depression among primary caregivers, measured using the Patient Health Questionnaire‐9 (PHQ‐9). Depression severity was categorized according to PHQ‐9 scoring, and participants meeting the study cut‐off were classified as having depression.

### 2.7 Quality Control

Quality control measures were applied throughout the study to ensure the accuracy, consistency, and completeness of the data. The study tools and procedures were pretested before the start of data collection, and research assistants were trained on participant recruitment, eligibility screening, informed consent, confidentiality, and administration of the study instruments. Standardized tools were used for all interviews, including the PHQ‐9, MSPSS, and DCFS. The principal investigator supervised data collection closely and reviewed completed questionnaires daily for completeness and internal consistency. Interviews were conducted in a private setting, and all records were labeled using unique study codes to maintain confidentiality and support accurate data management.

### 2.8 Data Management and Analysis

Data were collected using coded interviewer‐administered questionnaires, reviewed for completeness and consistency, and stored securely in locked file boxes. The data were entered into Microsoft Excel, cleaned, regularly backed up, and password‐protected before export to SPSS version 22.0 for analysis.

Descriptive statistics were used to summarize caregiver and patient characteristics. Categorical variables were presented as frequencies and percentages, while continuous variables were summarized using means with standard deviations or medians with interquartile ranges, depending on distribution. All analyses were two‐sided, and statistical significance was defined at *p* < 0.05.

The prevalence of depression was computed as the proportion of caregivers who screened positive for depression on the PHQ‐9 among all study participants, with corresponding 95% confidence intervals. Depression severity was further described as moderate, moderately severe, or severe according to PHQ‐9 scores. Factors associated with depression were explored using bivariable logistic regression, followed by multivariable logistic regression including variables with *p* < 0.20 at bivariable level and those considered clinically relevant. Crude and adjusted odds ratios with 95% confidence intervals were reported.

### 2.9 Human Ethics and Consent to Participate

Ethical approval for this study was obtained from the Kampala International University Research Ethics Committee (KIU‐REC) under approval number KIU‐2024‐332. Written informed consent was obtained from all participants before enrollment. The study was conducted in accordance with the Declaration of Helsinki, and confidentiality was maintained through the use of unique study codes and secure handling of study records.

## 3 Results

### 3.1 Characteristics of the Study Participants

A total of 376 primary caregivers participated in this study. More than half, 194 (51.6%), were aged 18–44 years, with a median age of 43.0 years (IQR: 30.3–54.0). The majority were female (62.8%), lived in rural areas (76.1%), and were married (55.9%). Primary education was the most common education level (43.4%), self‐employment was the most common occupation (45.2%), and most participants earned less than 200,000 UGX per month (72.3%). Parents were the largest caregiver group (42.0%). Most participants had DCFS stigma scores of 19.0–28.0 (64.1%) and MSPSS scores of 24.0–51.0 (62.2%) (Table 1).

**Table 1:** Characteristics of caregivers of individuals with severe mental illness (SMI) (*N* = 376).

Characteristics	Frequency (*N* = 376)	Percentages (%)
Age (years)	Median = 43.0, interquartile range = 30.3–54.0	
18–44	194	51.6
45–64	158	42.0
65+	24	6.4
Sex		
Male	140	37.2
Female	236	62.8
Residence		
Rural	286	76.1
Urban	90	23.9
Marital status		
Married	210	55.9
Single	66	17.6
Divorced/separated	51	13.6
Widow	49	13.0
Education level		
None	24	6.4
Primary	163	43.4
Secondary	123	32.7
Tertiary	66	17.6
Employment		
Unemployed	166	44.1
Self employed	170	45.2
Employed	40	10.6
Monthly income (UGX)		
< 200,000	272	72.3
200,000–500,000	81	21.5
> 500,000	23	6.1
Relationship		
Spouse	29	7.7
Sibling	94	25.0
Child	53	14.1
Parent	158	42.0
Other (friend or other relative)	42	11.2
Past mental illness		
No	331	88.0
Yes	45	12.0
DCFS stigma score	Median 23.0, interquartile range = 19.0–28.0	
< 19.0	62	16.5
19.0–28.0	241	64.1
> 28.0	73	19.4
MSPSS support score	Median = 44.0, interquartile range = 24.0–51.0	
< 24.0	52	13.8
24.0–51.0	234	62.2
> 51.0	90	23.9

Abbreviations: DCFS, devaluation of consumer families scale; MSPSS, multidimensional scale of perceived social support; UGX, Ugandan Shilling.

### 3.2 Characteristics of Patients Being Cared For

Regarding the characteristics of the patients being cared for, most were aged 18–44 years, 261 (69.4%). Slightly more than half were male, 189 (50.3%), and the largest proportion were single, 158 (42.0%). Most patients had attained primary education, 166 (44.1%), were unemployed, 286 (76.1%), and earned less than 200,000 UGX per month, 321 (85.4%). The most common diagnosis was bipolar affective disorder, reported in 166 (44.1%) patients. Most patients had no suicidal ideation, 323 (85.9%), had experienced two or more previous admissions, 223 (59.3%), and were compliant with medication, 195 (51.9%) (Table 2).

**Table 2:** Characteristics of patients being cared for.

Characteristics	Frequency (*N* = 376)	Percentages (%)
Age (years)	Median = 32.0, interquartile range = 23.0–42.0	
< 18	32	8.5
18–44	261	69.4
45–64	70	18.6
65+	13	3.5
Sex		
Male	189	50.3
Female	187	49.7
Marital status		
Married	107	28.5
Single	158	42.0
Divorced/separated	79	21.0
Widow	32	8.5
Education level		
None	10	2.7
primary	166	44.1
Secondary	141	37.5
Tertiary	59	15.7
Employment		
Unemployed	286	76.1
Self employed	72	19.1
Formal employed	18	4.8
Monthly income (UGX)		
< 200,000	321	85.4
200,000–500,000	37	9.8
> 500,000	18	4.8
Diagnosis		
Schizophrenia	90	23.9
Bipolar affective disorder	166	44.1
Major depression	56	14.9
Substances use disorder	64	17.0
Suicidal		
No	323	85.9
Yes	53	14.1
Admissions	Median = 2.0, interquartile range = 1.0–3.0	
None	26	6.9
One	127	33.8
Two and above	223	59.3
Medicine compliance		
No	181	48.1
Yes	195	51.9

Abbreviations: DCFS, devaluation of consumer families scale; MSPSS, multidimensional scale of perceived social support; UGX, Uganda shillings.

### 3.3 Prevalence and Patterns of Depression Among Caregivers of Individuals With SMI in Uganda (*N* = 376)

**Figure 1:**
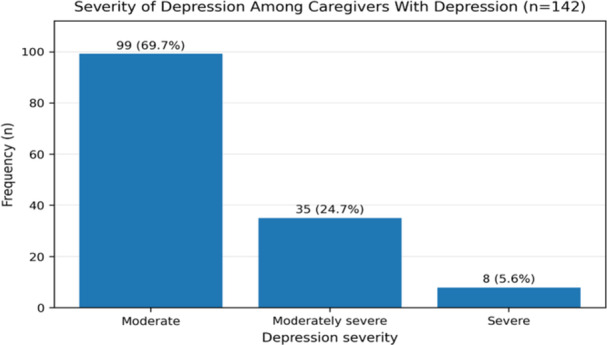
Prevalence and patterns of depression among caregivers of individuals with severe mental illness (SMI) in Uganda.

Among the 376 caregivers enrolled in the study, 142 had depression, corresponding to a prevalence of 37.8% (95% CI: 32.7%–42.8%). Among caregivers with depression, 99 (69.7%) had moderate depression, 35 (24.7%) had moderately severe depression, and 8 (5.6%) had severe depression (Figure 1).

### 3.4 Factors Associated With Depression Among Caregivers of Individuals With SMI in Uganda (*N* = 376)

At bivariable analysis, caregiver age, residence, marital status, relationship to the patient, perceived stigma score, perceived social support score, patient age, patient marital status, diagnosis, number of previous admissions, and medication compliance met the pre‐specified criterion of *p *< 0.20 for inclusion in the multivariable logistic regression model (Tables 3 and 4). After adjustment for potential confounders, perceived stigma, perceived social support, patient age, and patient diagnosis remained independently associated with caregiver depression. Caregivers with high perceived stigma had markedly higher odds of depression than those with low perceived stigma (aOR = 10.29, 95% CI: 3.16–33.50), and caregivers with low perceived social support had similarly increased odds compared with those reporting high support (aOR = 10.51, 95% CI: 2.95–37.45). Caregivers of patients aged 18–44 years and those caring for patients aged ≥ 65 years were also more likely to have depression than caregivers of patients aged < 18 years, with adjusted odds ratios of 4.20 (95% CI: 1.30–13.58) and 8.38 (95% CI: 1.50–46.89), respectively. In addition, caring for a patient with bipolar affective disorder was associated with lower odds of depression than caring for a patient with schizophrenia (aOR = 0.46, 95% CI: 0.21–0.98), whereas caring for a patient with major depression was associated with increased odds of caregiver depression (Table 5).

**Table 3:** Bivariable analysis of the characteristics of caregivers of individuals with severe mental illness (SMI) in Uganda (*N* = 376).

Characteristics	No depression, *N* = 234	Depression, *N* = 142	Bivariate analysis		
			cOR	95% CI	*p* value
Age (years)					
18–44	130 (55.6)	64 (45.1)	Ref		
45–64	86 (36.8)	72 (50.7)	1.701	1.103–2.622	**0.016**
65+	18 (7.7)	6 (4.2)	0.677	0.256–1.788	0.431
Sex					
Male	92 (39.3)	48 (33.8)	Ref		
Female	142 (60.7)	94 (66.2)	1.269	0.821–1.961	0.284
Residence					
Rural	172 (73.5)	114 (80.3)	1.468	0.886–2.432	**0.137**
Urban	62 (26.5)	28 (19.7)	Ref		
Marital status					
Married	136 (58.1)	74 (52.1)	Ref		
Single	47 (20.1)	19 (13.4)	0.743	0.406–1.358	0.334
Divorced/separated	30 (12.8)	21 (14.8)	1.286	0.688–2.404	0.430
Widow	21 (9.0)	28 (19.7)	2.450	1.302–4.613	**0.005**
Education level					
None	15 (6.4)	9 (6.3)	0.984	0.375–2.582	0.974
Primary	108 (46.2)	55 (38.7)	0.835	0.461–1.513	0.552
Secondary	70 (29.9)	53 (37.3)	1.242	0.673–2.290	0.488
Tertiary	41 (17.5)	25 (17.6)	Ref		
Employment					
Unemployed	128 (54.7)	38 (26.8)	0.617	0.290–1.311	0.209
Self employed	79 (33.8)	91 (64.1)	2.392	1.156–4.950	0.219
Formal employed	27 (11.5)	13 (9.2)	Ref		
Monthly income (in UGX)					
< 200,000	163 (69.7)	109 (76.8)	2.407	0.868–6.676	0.291
200,000–500,000	53 (22.6)	28 (19.7)	1.902	0.638–5.665	0.248
> 500,000	18 (7.7)	5 (3.5)	Ref		
Relationship					
Spouse	17 (7.3)	12 (8.5)	2.259	0.811–6.295	**0.119**
Sibling	59 (25.2)	35 (24.6)	1.898	0.833–4.328	**0.127**
Child	37 (15.8)	16 (11.3)	1.384	0.551–3.476	0.489
Parent	89 (38.0)	69 (48.6)	2.481	1.141–5.393	**0.022**
Other (friend or other relative)	32 (13.7)	10 (7.0)	Ref		
Past mental illness					
No	205 (87.6)	126 (88.7)	Ref		
Yes	29 (12.4)	16 (11.3)	0.898	0.469–1.719	0.745
DCFS stigma score					
< 19.0	51 (21.8)	11 (7.7)	Ref		
19.0–28.0	168 (71.8)	73 (51.4)	2.015	0.993–4.086	**0.052**
> 28.0	15 (6.4)	58 (40.8)	17.927	7.554–22.544	**< 0.001**
MSPSS support score					
< 24.0	7 (3.0)	45 (31.7)	29.732	11.357–77.835	**< 0.001**
24.0–51.0	153 (65.4)	81 (57.0)	2.449	1.339–4.478	**0.004**
> 51.0	74 (31.6)	16 (11.3)	Ref		

*Note:* Bivariable analysis: bold are *p* value < 0.2.

Abbreviations: CI, confidence interval; cOR, crude odds ratio; DCFS, devaluation of consumer families scale; MSPSS, multidimensional scale of perceived social support; UGX, Ugandan Shilling.

**Table 4:** Bivariable analysis of the characteristics of patients being cared for (*n* = 376).

Characteristic	No depression, *N* = 234	Depression, *N* = 142	Bivariate analysis		
			cOR	95% CI	*p* value
Age (years)					
< 18	25 (10.7)	7 (4.9)	Ref		
18–64	204 (87.2)	127 (89.4)	2.223	0.934–5.291	**0.071**
65+	5 (2.1)	8 (5.6)	5.714	1.414–23.097	**0.014**
Marital status					
Married/cohabiting	78 (33.3)	29 (20.4)	Ref		
Single	90 (38.5)	68 (47.9)	2.032	1.196–3.453	**0.009**
Divorced/separated	47 (20.1)	32 (22.5)	1.831	0.986–3.402	**0.056**
Widow	19 (8.1)	13 (9.2)	1.840	0.807–4.196	**0.147**
Education level					
None	4 (1.7)	6 (4.2)	1.904	0.486–7.459	0.355
Primary	125 (53.4)	41 (28.9)	0.416	0.223–1.776	0.206
Secondary	72 (30.8)	69 (48.6)	1.216	0.660–2.241	0.530
Tertiary	33 (14.1)	26 (18.3)	Ref		
Employment					
Unemployed	189 (80.8)	97 (68.3)	0.513	0.197–1.335	0.271
Self employed	36 (15.4)	36 (25.4)	1.000	0.356–2.809	1.000
Employed	9 (3.8)	9 (6.3)	Ref		
Monthly income (UGX)					
< 200,000	200 (85.5)	121 (85.2)	2.117	0.681–6.581	0.295
200,000–500,000	20 (8.5)	17 (12.0)	2.975	0.823–10.760	0.296
> 500,000	14 (6.0)	4 (2.8)	Ref		
Diagnosis					
Schizophrenia/schizoaffective disorder	52 (22.2)	38 (26.8)	Ref		
Bipolar affective disorder	131 (56.0)	35 (24.6)	0.366	0.209–0.640	**< 0.001**
Major depression	15 (6.4)	41 (28.7)	3.740	1.813–7.718	**< 0.001**
Substances use disorder	36 (15.4)	28 (19.7)	1.064	0.557–2.033	0.850
Admissions					
None	23 (9.8)	3 (2.1)	Ref		
One	87 (37.2)	40 (28.2)	3.525	1.000–12.427	**0.050**
Two and above	124 (53.0)	99 (69.7)	6.121	1.786–20.979	**0.004**
Medicine compliance					
No	93 (39.7)	88 (62.0)	2.471	1.610–3.791	**< 0.001**
Yes	141 (60.3)	54 (38.0)	Ref		

*Note:* Bivariable analysis: bold are *p* value < 0.2.

Abbreviations: CI, confidence interval; cOR, crude odds ratio; UGX, Ugandan Shilling.

**Table 5:** Multivariable analysis of factors associated with depression among caregivers of individuals with severe mental illness (SMI) in Uganda (N = 376).

Characteristics	Bivariate analysis			Multivariate analysis		
	cOR	95% CI	*p* value	aOR	95% CI	*p* value
Age (years)						
18–44	Ref			Ref		
45–64	1.701	1.103–2.622	0.016	1.021	0.573–1.819	0.944
65+	0.677	0.256–1.788	0.431	0.264	0.070–1.098	0.051
Residence						
Rural	1.468	0.886–2.432	0.137	1.246	0.639–2.432	0.519
Urban	Ref			Ref		
Marital status						
Married	Ref			Ref		
Single	0.743	0.406–1.358	0.334	0.721	0.305–1.703	0.455
Divorced/separated	1.286	0.688–2.404	0.430	0.826	0.334–2.039	0.678
Widow	2.450	1.302–4.613	0.005	1.989	0.764–5.179	0.159
Relationship						
Spouse	2.259	0.811–6.295	0.119	0.882	0.167–4.647	0.882
Sibling	1.898	0.833–4.328	0.127	0.979	0.339–2.829	0.968
Child	1.384	0.551–3.476	0.489	0.732	0.224–2.390	0.605
Parent	2.481	1.141–5.393	0.022	0.578	0.184–1.814	0.347
Other (friend or other relative).	Ref			Ref		
DCFS stigma score						
< 19.0	Ref			Ref		
19.0–28.0	2.015	0.993–4.086	0.052	2.419	0.994–5.888	0.052
**> 28.0**	**17.927**	**7.554–42.544**	**< 0.001**	**10.294**	**3.163–23.500**	**< 0.001**
MSPSS support score						
**< 24.0**	**29.732**	**11.35–77.34**	**< 0.001**	**10.513**	**2.951–14.449**	**< 0.001**
24.0–51.0	2.449	1.339–4.478	0.004	1.652	0.756–3.610	0.208
> 51.0	Ref			Ref		
Age (years) (patient)						
< 18	Ref			Ref		
**18–44**	**2.561**	**1.069–6.134**	**0.035**	**4.195**	**1.296–13.576**	**0.017**
45–64	1.236	0.457–3.343	0.676	2.606	0.709–9.576	0.149
**65** +	**5.714**	**1.414–23.097**	**0.014**	**8.381**	**1.498–14.891**	**0.016**
Marital status (patient)						
Married	Ref			Ref		
Single	2.032	1.196–3.453	0.009	1.920	0.814–4.529	0.136
Divorced/separated	1.831	0.986–3.402	0.056	1.195	0.445–3.207	0.723
Widow	1.840	0.807–4.196	0.147	2.149	0.661–6.984	0.203
Diagnosis						
Schizophrenia	Ref			Ref		
**Bipolar affective disorder**	**0.366**	**0.209–0.640**	**< 0.001**	**0.457**	**0.213–0.977**	**0.044**
**Major depression**	**3.740**	**1.813–7.718**	**< 0.001**	**7.453**	**2.759–9.135**	**< 0.001**
Substances use disorder	1.064	0.557–2.033	0.850	0.691	0.274–1.744	0.434
Number of admissions						
None	Ref			Ref		
One	3.525	1.000–12.427	0.050	2.625	0.545–12.651	0.229
Two and above	6.121	1.786–20.979	0.004	2.872	0.625–13.204	0.175
Medicine compliance						
No	2.471	1.610–3.791	< 0.001	1.566	0.819–2.994	0.175
Yes	Ref			Ref		

*Note:* Multivariable analysis: bold are *p* value < 0.05.

Abbreviations: aOR, adjusted odds ratio; CI, confidence interval; DCFS, devaluation of consumer families scale, MSPSS, multidimensional scale of perceived social support; UGX, Ugandan Shilling.

## 4 Discussion

The prevalence of depression among primary caregivers in this study was 37.8%, indicating that more than one in three caregivers experienced depressive symptoms. It is higher than the 19.0% reported among caregivers of adults with severe mental illness by Derajew et al. in southwest Ethiopia [16], and higher than the 18.3% reported among primary caregivers of individuals with mental illness by Jeyagurunathan et al. in Singapore [17]. However, it is lower than the 57.6% reported among caregivers of children and adolescents with mental illness by Minichil et al. in Addis Ababa [18]. It is also close to the 33.1% reported among caregivers of patients with severe mental illness by Munie et al. in northwest Ethiopia [19], and the 39.4% prevalence of common mental disorder reported among caregivers of adults with mental illness by Nigussie et al. in eastern Ethiopia [20]. These contrasts suggest that the burden observed in our study is high, regionally plausible, and consistent with the wider literature showing substantial psychological morbidity among family caregivers of people with mental illness. Differences across studies are likely to reflect variation in caregiver populations, patient age groups, outcome definitions, screening tools, and local service contexts.

The severity pattern in our study also deserves attention. Among caregivers with depression, 69.7% had moderate depression, 24.7% had moderately severe depression, and 5.6% had severe depression. This shows that the burden was not limited to mild emotional distress. Nearly one‐third of affected caregivers had moderately severe or severe symptoms, which may require closer psychological assessment and more structured support. Direct comparative studies reporting PHQ‐9 severity distributions in caregivers of people with severe mental illness are limited. However, Derajew et al. reported that, in their full caregiver sample, 11.3% had moderate depression, 3.5% had moderately severe depression, and 4.2% had severe depression [16]. Although the denominator used in that study differs from ours, both studies suggest that moderate depressive symptoms account for the largest share of caregiver depression.

The present findings are also consistent with the Ugandan caregiving context. Ndikuno et al. found that quality of life among caregivers of patients with severe mental illness in Uganda was generally poor, while Verity et al. described caregiving for persons with severe mental illness in Uganda as emotionally demanding and carried out with limited formal support [21, 22]. Although those studies did not measure depression as the main outcome, they support the interpretation that caregiving in Ugandan psychiatric settings is psychologically burdensome. Our study extends this evidence by showing that depressive symptoms are not only present but also common.

A major finding of this study was the strong association between high perceived stigma and caregiver depression. Caregivers with higher stigma scores had markedly greater odds of depression than those with lower scores. This is consistent with findings from Egypt, where depressive disorders among caregivers of patients with schizophrenia were closely related to perceived stigma and burden of care [23]. It is also in line with Ethiopian evidence showing that perceived stigma and common mental disorder frequently coexist among caregivers of adults with mental illness [20]. These findings suggest that stigma affects not only people living with mental illness, but also the family members who care for them. In practical terms, this means that anti‐stigma interventions should include caregivers as well as patients.

Low perceived social support was another strong correlate of caregiver depression and one of the largest effect sizes in the adjusted model. This finding is important because social support is potentially modifiable. In southwest Ethiopia, Derajew et al. found that lower perceived social support was associated with caregiver depression [16], while Minichil et al. similarly reported that poor social support was a predictor of depression among caregivers of children and adolescents with mental illness [18]. Nigussie et al. also found that poor social support was associated with common mental disorders among caregivers of adults with mental illness [20]. The present result points in the same direction. In settings where caregiving is largely family‐based, weak emotional, practical, and financial support may leave one caregiver carrying most of the burden alone, thereby increasing psychological distress.

We also found that patient age was independently associated with caregiver depression. Caregivers of patients aged 18–44 years and those caring for patients aged 65 years or older had higher odds of depression than caregivers of patients younger than 18 years. Direct comparative evidence on patient age as a predictor of caregiver depression in psychiatric settings remains limited, but the finding is clinically plausible. For younger adults, mental illness may interrupt expected roles in education, work, and family life, thereby increasing caregiver worry and uncertainty. For older patients, caregiving may be more difficult because of physical frailty, comorbidity, and greater dependence. In the Ugandan context, where caregiving often occurs with limited formal support, these age‐related demands may be especially important [22].

Patient diagnosis was also important. Compared with schizophrenia, caring for a patient with major depression was associated with substantially higher odds of caregiver depression, whereas caring for a patient with bipolar affective disorder was associated with lower odds. The higher odds observed for major depression may reflect the emotional burden of caring for someone with persistent low mood, functional impairment, hopelessness, and possible suicidality. Diagnosis‐related differences in caregiver distress have been described in other settings, although not always consistently. For example, Jeyagurunathan et al. showed that caregivers of individuals with mental illness experience significant psychological morbidity across diagnostic groups [17], while Semaan et al. similarly highlighted the substantial emotional and quality‐of‐life burden associated with caring for a mentally ill patient at home [24]. These findings suggest that diagnosis‐specific differences should be interpreted cautiously and within the clinical context of the study population.

Overall, the findings suggest that caregiver depression in this setting is shaped by both social vulnerability and patient‐related clinical factors. The strongest associations were observed for stigma and social support, indicating that the social environment of caregiving plays a central role in caregiver mental health. Because this was a cross‐sectional study, these findings should be interpreted as associations rather than causal pathways. Even so, they point to clear opportunities for practice, including routine caregiver screening in psychiatric clinics, caregiver‐focused psychoeducation, stigma‐reduction strategies, and stronger family and community support systems.

### 4.1 Strength and Limitations

This multicenter study provides comprehensive data on the predictors of depression among caregivers of individuals with SMI, offering a valuable baseline for future research in Uganda. However, the sample was drawn from two referral hospitals, which may limit the generalizability of the findings to other healthcare settings.

This study has several limitations. Because participants were recruited consecutively from two regional referral hospitals, the sample may overrepresent caregivers who are already engaged with specialized mental health services, which introduces the possibility of selection bias and may limit generalizability to caregivers in the broader community or lower‐level facilities. In addition, the cross‐sectional design precludes causal inference and does not allow temporal relationships between caregiver characteristics and depression to be established. Self‐reported psychosocial and caregiving variables may also have been affected by recall and social desirability bias.

## 5 Conclusions and Recommendations

This study revealed a high prevalence of depression among caregivers of individuals with severe mental illness in Uganda, surpassing findings from other African studies. The key factors contributing to caregiver depression included high perceived stigma, low social support, and both the age and diagnosis of the patient. Caregivers of younger and older patients experienced higher levels of depression. Additionally, caregivers of individuals with major depression were at significantly greater risk of depression than were those caring for individuals with other psychiatric conditions. Based on the study's findings, we recommend that healthcare providers screen for depressive symptoms among caregivers to address their mental health needs and enhance their ability to provide care. Additionally, psychoeducational sessions should be offered to both caregivers and patients in psychiatric units to increase understanding of mental illness and reduce stigma. Establishing community‐based support groups and networks is also crucial for providing ongoing emotional and practical assistance to caregivers.

## Author Contributions

**Florent Ishimwe:** investigation, validation, conceptualization, software, and methodology. **Marie Pascaline Sabine Ishimwe:** conceptualization, methodology, software, data curation, and formal analysis. **Maxwell Okello:** investigation, validation, and methodology. **Theodore Nteziyaremye:** investigation, validation, methodology, and software. **Alinaitwe Racheal:** investigation, validation, and software. **Usman Ibe Micheal:** investigation, methodology, validation, software, and formal analysis. **Joshua Muhumuza:** investigation, validation, and formal analysis. **Ildephonse Dushimirimana:** investigation, methodology, and validation. **Musinguzi Ronald:** investigation, validation, and software. **Abdirazak abdinazir:** investigation, funding acquisition, visualization, and validation. **Kiswezi Kazigo Ahmed:** investigation, visualization, validation, formal analysis, writing – review and editing. **Norbert Gumisiriza:** investigation, funding acquisition, supervision, data curation, and writing – original draft. **Theoneste Hakizimana:** conceptualization, methodology, formal analysis, supervision, writing – review and editing.

## Funding

The authors have nothing to report.

## Conflicts of Interest

The authors declare no conflicts of interest.

## Transparency Statement

The corresponding author, Theoneste Hakizimana, affirms that this manuscript is an honest, accurate, and transparent account of the study being reported; that no important aspects of the study have been omitted; and that any discrepancies from the study as planned (and, if relevant, registered) have been explained.

## Data Availability

The data sets used and analysed during the current study are available from the corresponding author upon reasonable request, and the data set used can be accessed with the permission of the corresponding author Dr. Theoneste Hakizimana (email: theonestehakizimana5@gmail.com).
